# Pay or prevent? Human safety, costs to society and legal perspectives on animal-vehicle collisions in São Paulo state, Brazil

**DOI:** 10.1371/journal.pone.0215152

**Published:** 2019-04-11

**Authors:** Fernanda Delborgo Abra, Beatriz Machado Granziera, Marcel Pieter Huijser, Katia Maria Paschoaletto Micchi de Barros Ferraz, Camilla Mansur Haddad, Roberta Montanheiro Paolino

**Affiliations:** 1 Forest Science Department, Luiz de Queiroz College of Agriculture, Piracicaba, SP, Brazil; 2 School of Forestry and Environmental Studies, Yale University, New Haven, CT, United States of America; 3 Western Transportation Institute, Montana State University, Bozeman, MT, United States of America; 4 Pontifical Catholic University of São Paulo, São Paulo, SP, Brazil; Universitat de Valencia, SPAIN

## Abstract

Direct road mortality and the barrier effect of roads are typically identified as one of the greatest threats to wildlife. In addition, collisions with large mammals are also a threat to human safety and represent an economic cost to society. We documented and explored the effects of animal-vehicle crashes on human safety in São Paulo State, Brazil. We estimated the costs of these crashes to society, and we summarized the legal perspectives. On average, the Military Highway Police of São Paulo reported 2,611 animal-vehicle crashes per year (3.3% of total crashes), and 18.5% of these resulted in human injuries or fatalities. The total annual cost to society was estimated at R$ 56,550,642 (US $ 25,144,794). The average cost for an animal-vehicle crash, regardless of whether human injuries and fatalities occurred, was R$ 21,656 (US $ 9,629). The Brazilian legal system overwhelmingly (91.7% of the cases) holds the road administrator liable for animal-vehicle collisions, both with wild and domestic species. On average, road administrators spent R$ 2,463,380 (US $ 1,005,051) per year compensating victims. The logical conclusion is that the Brazilian legal system expects road administrators to keep animals, both wild and domestic species, off the road. We suggest an improved coordination between the laws that relate to animal-vehicle collisions and human safety, and the process for environmental licenses that focusses on reducing collisions with wildlife and providing habitat connectivity. In addition, we suggest better management practices, raising awareness and social change with regard to abandoned domesticated animals including horses, cattle, and dogs. This should ultimately result in a road system with improved human safety, reduced unnatural mortality for both domestic and wild animal species, safe crossing opportunities for wildlife, and reduced monetary costs to society.

## Introduction

On a global scale, roads have important benefits for society as they allow for the transportation of people and goods. However, they also represent one of biggest threats to biodiversity [1], [2], [3]. For animals, the effects of roads and traffic are varied and range from habitat loss [4], direct mortality through collisions with vehicles [1], [5], barrier effects [6], [7] and a reduction in habitat quality in a zone adjacent to the road (e.g. noise, lights, pollution, visual disturbance) [8], [4], [9].

Animal-vehicle collisions are not only a biological conservation concern, but they are also a threat to human safety and the associated economic costs are high. The total number of large mammal-vehicle collisions (≥ 15 kg) per year has been estimated at one to two million in the United States and about 507,000 in Europe [10], [11], [12]. These types of collisions have increased substantially over the last few decades [11], [12]. While specific roads can be part of a research project or a citizen science project, most roads usually only have two sources for animal-vehicle collision data. These two sources each have their own terminology. One source is through law enforcement personnel who report on “animal-vehicle crashes”. Another source is through road maintenance personnel who inspect the roads on a regular basis, including the removal and reporting of “animal carcasses” on and immediately adjacent to the road. We applied this terminology throughout this manuscript; when we refer to crash data, this relates to data collected by law enforcement personnel, whilst carcass data refers to data collected by road maintenance personnel. The combination of these two data types (or other potential sources that include either “animal crash data” or animal carcass removal or observation data) we use the term “collision data”.

Animal-vehicle crashes account for 4–5% of all reported crashes in the United States [13], [12]. However, this percentage can be much higher in rural or natural areas with more abundant habitat and wildlife populations [14], [15]. For example, in Sweden, ungulate-vehicle-collisions accounted for over 60% of all road accidents reported to the police during the 1990s [16]. In the United States and Europe, most of the large wild mammal-vehicle collisions occur in the fall (October-December) with a smaller peak in the spring and early summer (May-June) [11], [12].

Large mammal-vehicle collisions predominantly occur in the dark, especially around dusk till midnight, and around dawn [11], [12]. In the United States and Canada, 2.8–9.7% of reported deer-vehicle crashes (*Odocoileus* sp.) resulted in human injuries, but the risk increased to 18–23% for larger species such as moose (*Alces americanus*) [17]. Similarly, the risk of human fatalities associated with an animal-vehicle collision also increased with the size of the animal; 0.0003% for deer and 0.004% for moose [17]. In the United States, large mammal-vehicle collisions were estimated to cause 211 human fatalities and 29,000 human injuries per year with associated costs between US$ 6–12 billion [10], [18]. In Europe, collisions with ungulates resulted in about 300 human fatalities and 30,000 human injuries per year, and over one billion dollars in vehicle repair costs [11].

In South America, little information is available on the threat that animal-vehicle collisions pose to human safety and the associated costs to society. However, in 2014, the Brazilian Federal Highway Police reported 3,174 animal-vehicle crashes on the Brazilian Federal Highway system [19]. Animal-vehicle crashes represented 1.9% of all reported crashes [19], of which 40.9% resulted in human injuries, and 2.6% resulted in human fatalities [19]. This suggests that the probability of an animal-vehicle crash resulting in a human injury or fatality is higher in Brazil than in North America. This may be related to potential differences in the size of the animal species involved, vehicle type, presence and use of seatbelts and airbags, road design characteristics, driving style, and response time of emergency services. Differences in reporting thresholds for animal-vehicle crashes between the United States and Brazil may also be a reason for reports of animal-vehicle crashes

In the United States and Canada, it is typically the more severe animal-vehicle collisions that are included in the crash database. Severe collisions typically include human injuries or fatalities, an estimate of at least US$ 1000 in vehicle repair costs, or a disabled vehicle that needs to be towed [16]. In contrast, in Brazil, for an animal-vehicle collision to be included in the crash database, a collision must be reported to law enforcement personnel, either by the occupants of the vehicle involved in the collision, or by people who happen to pass by the scene of a collision. There are no minimum damage thresholds for an animal-vehicle collision in the crash database in Brazil, which suggests that the thresholds for an animal-vehicle collision to be included in a crash database are lower in Brazil than in the United States and Canada.

Animal species that have been documented as possible greatest concern to human safety on highways in Brazil are lowland tapir (*Tapirus terrestris*), capybara (*Hydrochoerus hydrochoeris*), and large domesticated species such as cattle (*Bos taurus*) and horses (*Equus caballus*) [20], [21], [22], Pedro Romanini, Agência de Transporte do Estado de São Paulo (ARTESP), pers. comm., 2018). The vehicle repair costs associated with capybara-vehicle collisions have been estimated at about R$ 2,885 (US $ 1,418, in 2012) [20], [23]. Collisions with larger species such as lowland tapir (~200–300 kg), cattle (~250–750 kg) and horses (~300–500 kg), are likely to present a greater threat to human safety and have higher associated costs.

In this paper we document and explore the effects of animal-vehicle crashes on human safety in São Paulo State in Brazil. In addition, we estimate the costs of these animal-vehicle crashes to society, and we summarize the legal perspectives with regard to liability and associated financial compensation for animal-vehicle collisions.

## Materials and methods

### Study area

São Paulo State is located in the southeast of Brazil, South America. In general, the region has a tropical climate with seasonal differences in temperature and precipitation (22–28 ºC on average; 1450–2050 mm precipitation annually) [24]. In addition, there are regional differences in weather patterns because of elevation, slope and distance to the Atlantic Ocean. These differences result in varying vegetation cover from the savannah in the interior to the Atlantic forest along the coast [24]. São Paulo State is home to approximately 44 million people, 21,6% of the total population in Brazil.

During the 20th century, São Paulo State experienced rapid land use change due to an expansion of urban areas, human population growth and agricultural development, as well as an expansion of existing road networks (33% increase in length between 1988 to 2013) [25]. São Paulo State has one of the highest road densities in Brazil; 0.8 km roads/km2 (37,000 km paved roads and 163,000 unpaved roads) [26].

This process has resulted in severe natural habitat loss and fragmentation of two of the world’s biodiversity hotspots, the Brazilian Savanah and Atlantic Forest [27]. In São Paulo State only 17,5% of the Atlantic Forest and the Brazilian Savanah remain [28]. Currently, the land use in São Paulo State is dominated by agriculture (~59%—mostly sugar cane, pastures, and eucalyptus plantations) and urban areas (~5%) [26].

### Animal-vehicle crash numbers and temporal patterns

Crash data gathered over an eleven-year period (2003 to 2013) was obtained from the Military Highway Police of São Paulo State (Polícia Militar do Estado de São Paulo—PMRSP). These data comprise a total of 37,000 km of major state highways managed by the transportation agency of São Paulo (Departamento de Estradas de Rodagem (DER)) (~30,500 km) and toll road companies (~ 6,500 km).

The roads managed by DER usually have two lanes (each lane is ~3.5 m wide), narrow or non-existent clear zones, posted legal speed limit varying between 30 to 110 km/h, frequent absence of street lights, and relatively slow and poor medical assistance. In comparison, toll roads tend to be major four lane highways that have been reconstructed over the last few decades [29]. These highways tend to have wide clear zones, guard rails and median barriers (e.g. concrete Jersey median barriers), street lights in selected areas, and relatively fast and modern medical and mechanical assistance provided by the toll road companies. The traffic volume on toll roads is typically higher than on DER roads, and the posted legal speed limit usually varies between 80–120 km/h [29].

For animal-vehicle crashes, the following parameters were recorded: date, time, municipality, road name or number, road administrator (toll road company or DER), location based on km post, vehicle type, number of vehicles involved in the crash, number of humans with minor or severe injuries, and number of human fatalities. The crash data did not include information on the animal species involved, nor whether the species was wild or domesticated.

The vehicle type categories included bicycle, motorcycle, passenger car, pick-up truck, truck, bus and other (semi-truck).The victim categories included: 1) no victims: driver and potential occupants of the vehicle do not present physical injuries or unconsciousness; 2) minor injuries: minor physical injuries or post-traumatic stress disorder; 3) severe injuries: Incapacitating physical injuries, unconsciousness and, 4) fatalities: driver or occupants of the vehicle die on site or later because of the crash.

For a crash to be included in the PMRSP database, the crash had to be reported to law enforcement personnel. Vehicle occupants involved in a crash, or people who happen to pass by the scene of a crash can make a request by phone to law enforcement for assistance and for issuing a police report of the crash. Each crash is recorded only once; there is no possibility for duplicates for the crash records collected by the PMRSP. In addition, the reporting effort remained the same throughout the study period. Note that the number of crashes in the PMRSP dataset is only a small portion of the number of animal carcasses that are removed from the highways by road maintenance personnel because most of the animal-vehicle collisions are not reported to PMRSP. From 2003 to 2013, the total number of animal-vehicle crashes (domestic and wild species combined) in the PMRSP dataset was 28,724 (average of 2,611 animal-vehicle crashes per year), whilst the toll road companies reported 32,258 carcasses (average of 3,584 carcasses per year) of wild mammal species [29] between 2005–2013 (1.4 times that of the reported crashes by the PMRSP). Note the toll roads only covered 17.6% of the total road length covered by the PMRSP, 6,500 km of toll roads out of a total of 37,000 km of roads). These carcass removal data were obtained by toll road maintenance personnel that checked the general road condition, including the potential presence of animal carcasses, at least once every three hours, on a daily basis. This illustrates that not every animal that is hit by a vehicle results in substantial vehicle damage or human injuries and fatalities, nor does it result in a request for police assistance, or a record in the crash database of the PMRSP.

### Data analyses

#### Animal-vehicle crash numbers and temporal patterns

We conducted an exploratory analysis and calculated the percentage of animal-vehicle crashes for all reported crashes in São Paulo State based on the PMRSP crash database. We calculated the total number of animal-vehicle crashes, the number of crashes with human injuries and fatalities, and the frequency distribution for both time of day and month, by road administrator (managed by either toll road companies or DER), and by vehicle type. We ran two linear regression analyses to investigate potential changes in the total number of crashes (all types of crashes) and the number of animal-vehicle crashes for the data period (2003–2013).

#### Costs of animal-vehicle crashes to society

Based on the animal-vehicle crashes in the PMRSP database and the IPEA report “Traffic Accidents along Brazilian Federal Roads: Characterization, Trends and Costs to Society” [19], we calculated the costs associated with each of the reported animal-vehicle crashes between 2003–2013.

The costs of an animal-vehicle crash in the IPEA report is based on three cost components; costs associated with people, vehicles, and public service and damage to public property (Table 1). We summarized the costs for each component by vehicle type in S1 Table.

**Table 1:** Description of components for each category of costs [19].

People costs	Vehicle costs	Public service and public property costs
Pre-hospital care	Vehicle removal	Crash assistance
Hospital care	Material damage	Property damage to road or roadside objects
Post-hospital care	Commercial cargo loss (if applicable)	-
Loss of productivity		-
Transport of victim	-	-

For crashes without human injuries or fatalities, the IPEA report [19] provides cost estimates based on associated cost with people, vehicles, and public service and public property damage. For example, even when there is no victim with apparent injuries, there is still a cost associated when there is a loss of productivity in cases of psychological trauma that may require professional treatment. Furthermore, vehicle costs include vehicle removal and vehicle repair costs, and costs associated with the loss of commercial cargo. Public service and public property damage relate to crash assistance by law enforcement, road management, and road assistance personnel, and repair costs to the road or roadside objects such as signs or guard rails. We calculated the costs of crashes without victims based on the values summarized in S2 Table.

For crashes with one or more human injuries or human fatalities, and crashes that involved multiple vehicles, the data did not specify what vehicle type the human injuries and fatalities related to. Since the associated costs depend on the vehicle type (see S3 Table) [19], we allocated the costs associated with human injuries and fatalities to the smallest vehicle type involved in the crash (e.g. bicycle < motorcycle < car < pick-up truck < truck < bus < other). This resulted in a conservative (i.e. low–see S2 Table) cost estimate of the crash as we erred towards the smaller vehicle types which have lower costs associated with a crash than the larger vehicle types. If there were multiple human injuries or fatalities, we allocated potential human fatalities and human injuries to the smallest vehicle type, up to the maximum number of occupants for that type of vehicle (see S3 Table).

Additional human fatalities were allocated to the second smallest vehicle and so forth. For example, if the crash included an animal, and both a motorcycle and a passenger car, and if the crash resulted in one human fatality and two humans with severe injuries, we allocated the costs associated with the human fatality and one human with severe injuries to the motorcycle (the maximum number of occupants for a motorcycle was set at two) and the costs associated with the second human with severe injuries to the passenger car (the maximum number of occupants for a passenger car was set at five). The costs for each type of accident with or without human injuries and fatalities was based on the values summarized in S2 Table.

#### Legal perspectives and financial compensation

We examined records of the Court of Appeal of the State of São Paulo (TJSP–Tribunal de Justiça do Estado de São Paulo—2° instância) and selected court cases that related to animal-vehicle collisions. We investigated which laws and legal perspectives were applied in court cases where plaintiffs requested financial compensation for the damage and losses associated with an animal-vehicle collision.

Though other courts in São Paulo State (e.g. Small Claims Court (Juizado Especial de Pequenas Causas) and the 1^st^ Instance of the Court of the State of São Paulo (Tribunal de Justiça do Estado de Sao Paulo– 1° instância)), may judge similar cases, we argue that TJSP´s decisions are most relevant for the purpose of this article because those case laws supersede decisions from the small claims court and the 1^st^ Instance Court, the arguments of the plaintiffs and defendants are better developed since they have already been processed through other courts, and we were able to access the position of the court with regard to where the legal responsibility lies for animal-vehicle collisions based on the laws in São Paulo State. In addition, the decisions by the judges of TJSP were available in electronic format which allowed us to select and investigate a large number of cases.

We searched the TJSP database with the following Portuguese keywords: “animal”, “acidente”, “rodovia” and “colisão” and identified 2,261 court cases. For a case to be included in our analyses, it had to meet the following criteria:

1. The collision occurred on a paved road;
2. The collision involved at least one vehicle and one animal (domesticated or wild species);
3. The road administrator (toll road company or DER) was the defendant;

These criteria resulted in 797 court cases, from which we created a database with the following parameters for each court case (if specified in the documents): year of the verdict, road administrator (managed by either a toll road company or DER), animal species or species group involved in the accident, verdict of the court, laws and regulations on which the verdict was based, and the total amount of R$ awarded to the plaintiffs. We calculated the number of cases and human fatalities per year and per road administrator type (i.e. toll road companies or DER). We also listed the animal species or species groups involved with the collisions and we summarized the costs in a box plot for species with at least 10 court cases.

For each court case we identified the total amount awarded by the court (if specified in the documents). The awarded amounts can consist of the following components: i) moral and mental suffering (impact on psychological, moral and intellectual mood, whether by offense to their honor, their privacy, intimacy, image, name or in their own physical body), ii) physical injuries and aesthetics (direct offense to the physical integrity of the human person), iii) material damage (damage caused to material property), and iv) loss of income (losses caused by the interruption of work and associated income).

In addition to these four components, the awarded amount can also include compensation for legal fees and a life pension (pensão vitalícia). Brazilian courts can award a life pension in cases where a person is disabled or killed because of the actions of another party (see S1 Text for further details). However, most verdicts do not specify amounts for the compensation for legal fees and a life pension. Instead they refer to the 1^st^ Instance of the Court of the State of São Paulo to calculate the amounts based on certain guidelines. Therefore, for our analyses, we excluded awarded amounts that related to legal fees and a life pension and, the awarded values we used for our analyses represent a very conservative estimate of the costs associated with animal-vehicle collisions. Whilst we recognize that this method of analysis has limitations, we believe that this does not influence the main points we make in this article; animal-vehicle collisions are a threat to human safety, there is an economic cost associated with these animal-vehicle collisions to society, and there is a legal perspective on the responsibility for these types of collisions.

## Results

### Animal-vehicle crash numbers and temporal patterns

**Fig 1:**
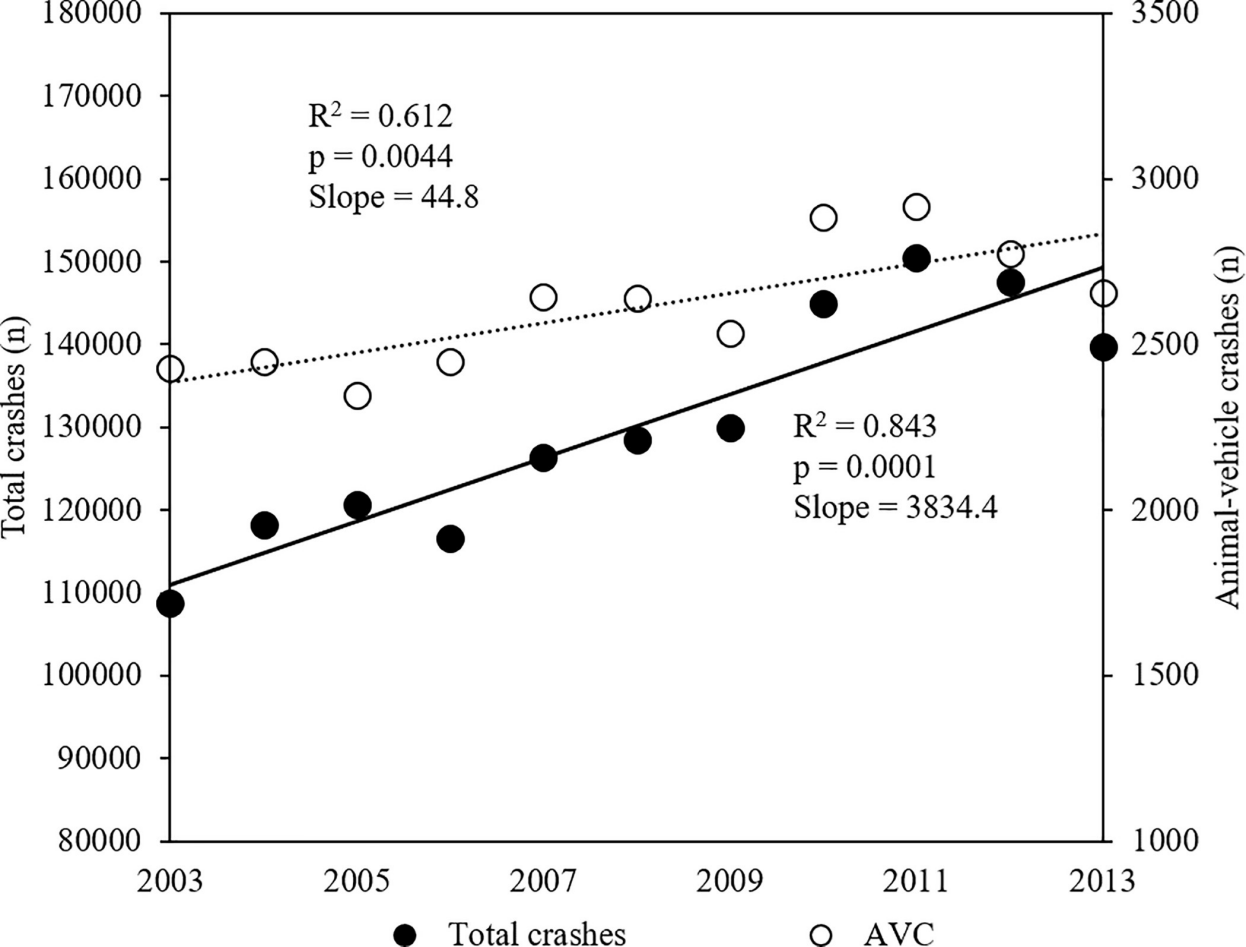
Results of linear regressions models. Dots represent the total number of crashes (all types) and animal-vehicle crashes (AVC) per year (from 2003–2013) on both private and public state roads in São Paulo State.

Over an eleven-year period (2003–2013), the PMRSP database included 889,797 records of crashes (all types) in São Paulo State, including 28,724 (3.3%) animal-vehicle crashes (Fig 1).

On average, there were 2,611 reported animal-vehicle crashes per year (minimum number 2,347, maximum number 2,916), resulting in an average of 483 crashes (18.5%) involving human injuries or fatalities. Based on the regression analyses there was an average increase of 3,834.4 total reported crashes per year (R^2^ = 0.84, p = 0.0001) and an average increase of 44.8 reported animal-vehicle crashes per year (R^2^ = 0.61, p = 0.004) (Fig 1). The crashes with human victims resulted in a yearly average of 531 humans with minor injuries, 116 humans with severe injuries, and 20 human fatalities.

**Fig 2:**
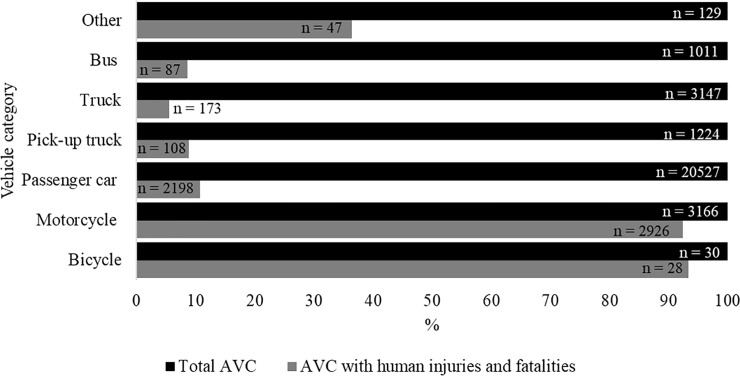
The total number of animal-vehicle crashes per vehicle type (set at 100%) and the percentage of those crashes with human injuries and fatalities on both private and public state roads in São Paulo State.

Animal-vehicle crashes with bicycles and motorcycles almost always resulted in human injuries and fatalities (> 90% of cases; Fig 2). There were 201 crashes with human fatalities, regardless of vehicle type. Motorcycles were most often involved with these fatal crashes (n = 94, 46.8% of all crashes with human fatalities). Crashes with passenger cars, pick-up trucks, trucks, and buses were much less likely to result in human injuries and fatalities (around 10%).

**Fig 3:**
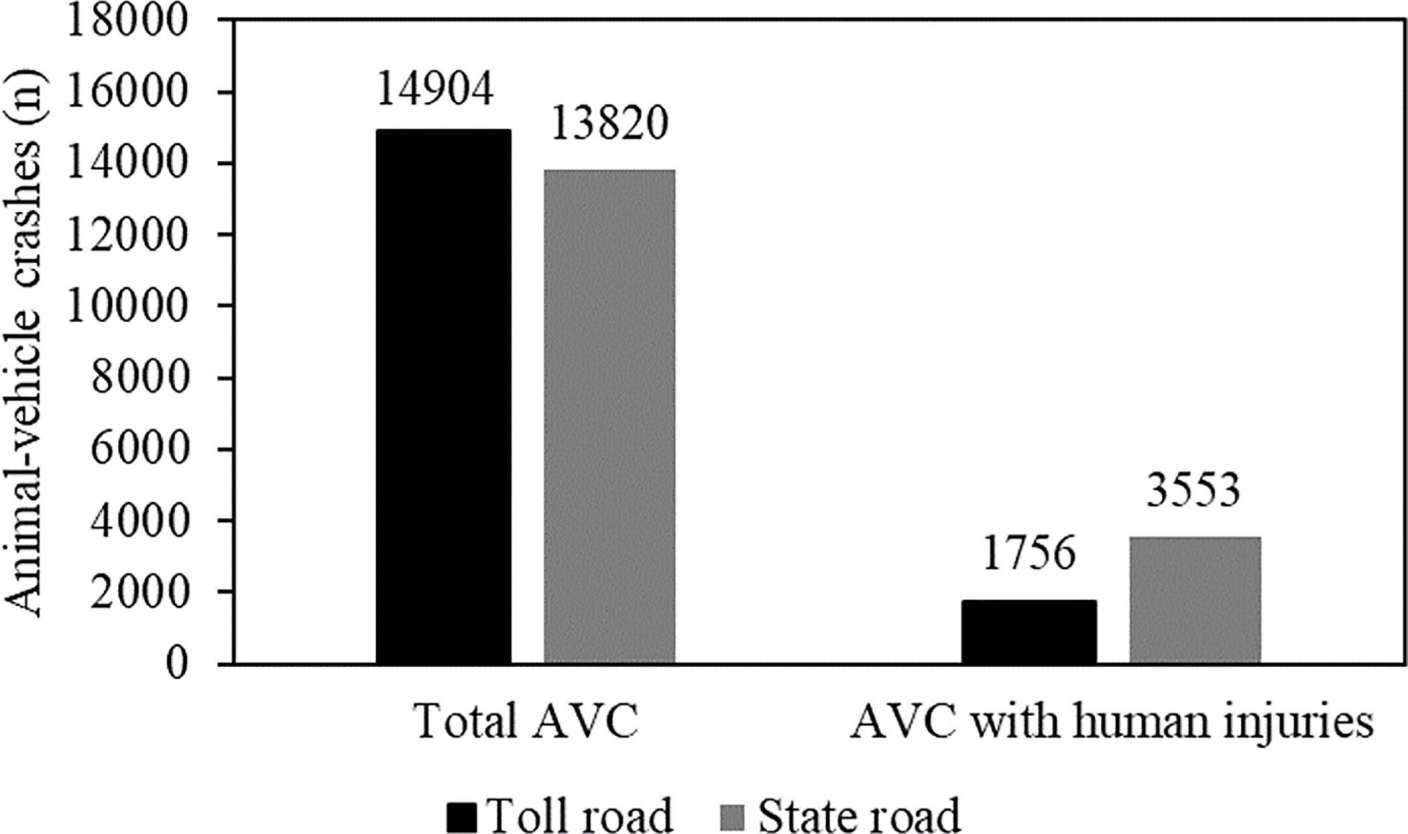
Total of number of animal-vehicle crashes and animal-vehicle crashes with human injuries and fatalities (2003–2013) on both private and public state roads in São Paulo State.

A total of 52% (n = 14,876) of the crashes were recorded on toll roads and 48% (n = 13,819) on state roads managed by DER. Though the total number of animal-vehicle crashes was similar for roads managed by toll road companies and DER, the number of animal-vehicle crashes with human injuries or fatalities on DER roads was more than double the number recorded along toll roads (Fig 3).

**Fig 4:**
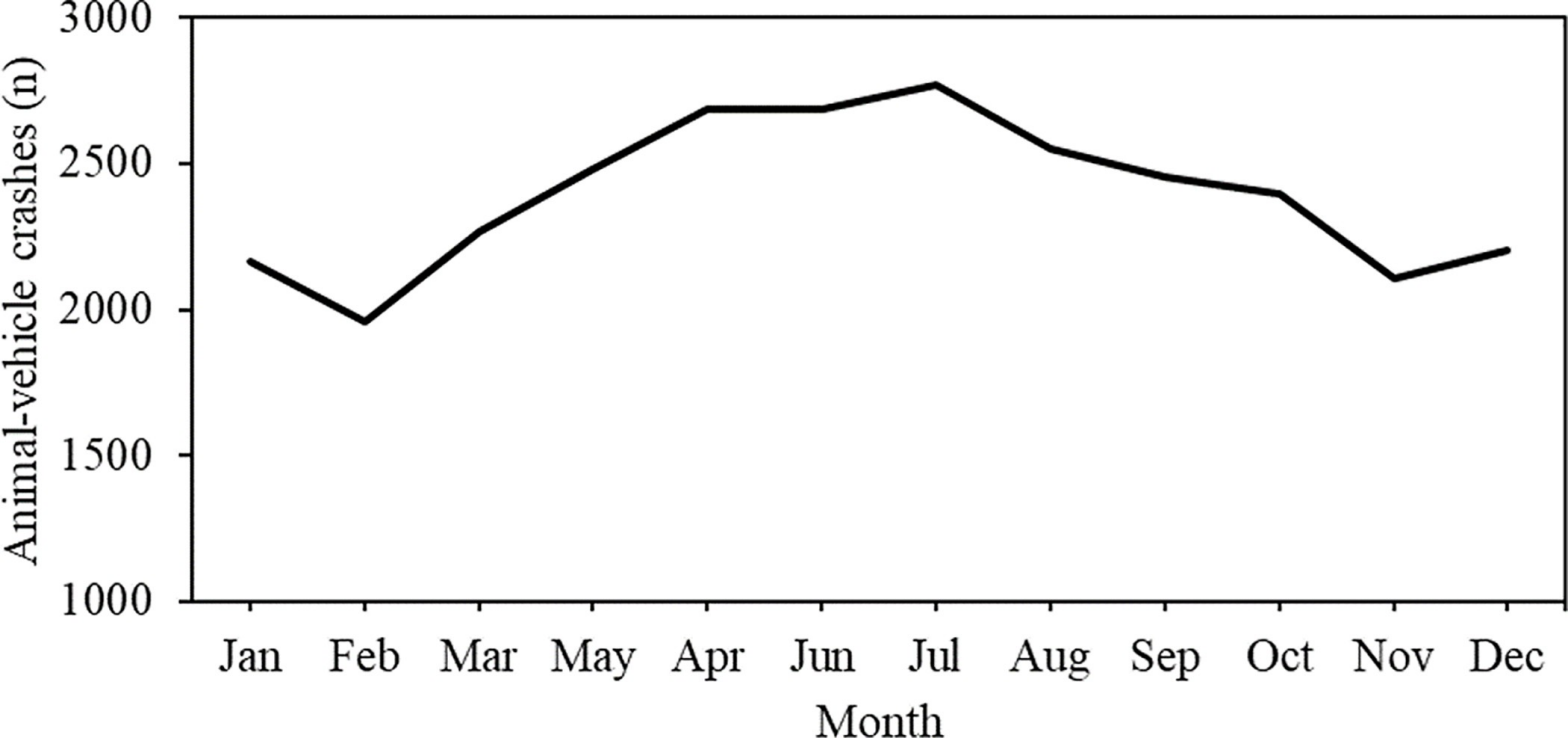
Total number of animal-vehicle crashes (2003–2013) per month on both private and public state roads in São Paulo State.

The number of animal-vehicle crashes showed some seasonal variation between the months of the year (Fig 4) with slightly fewer crashes during the wet season (October-March) compared to the dry season (April-September).

**Fig 5:**
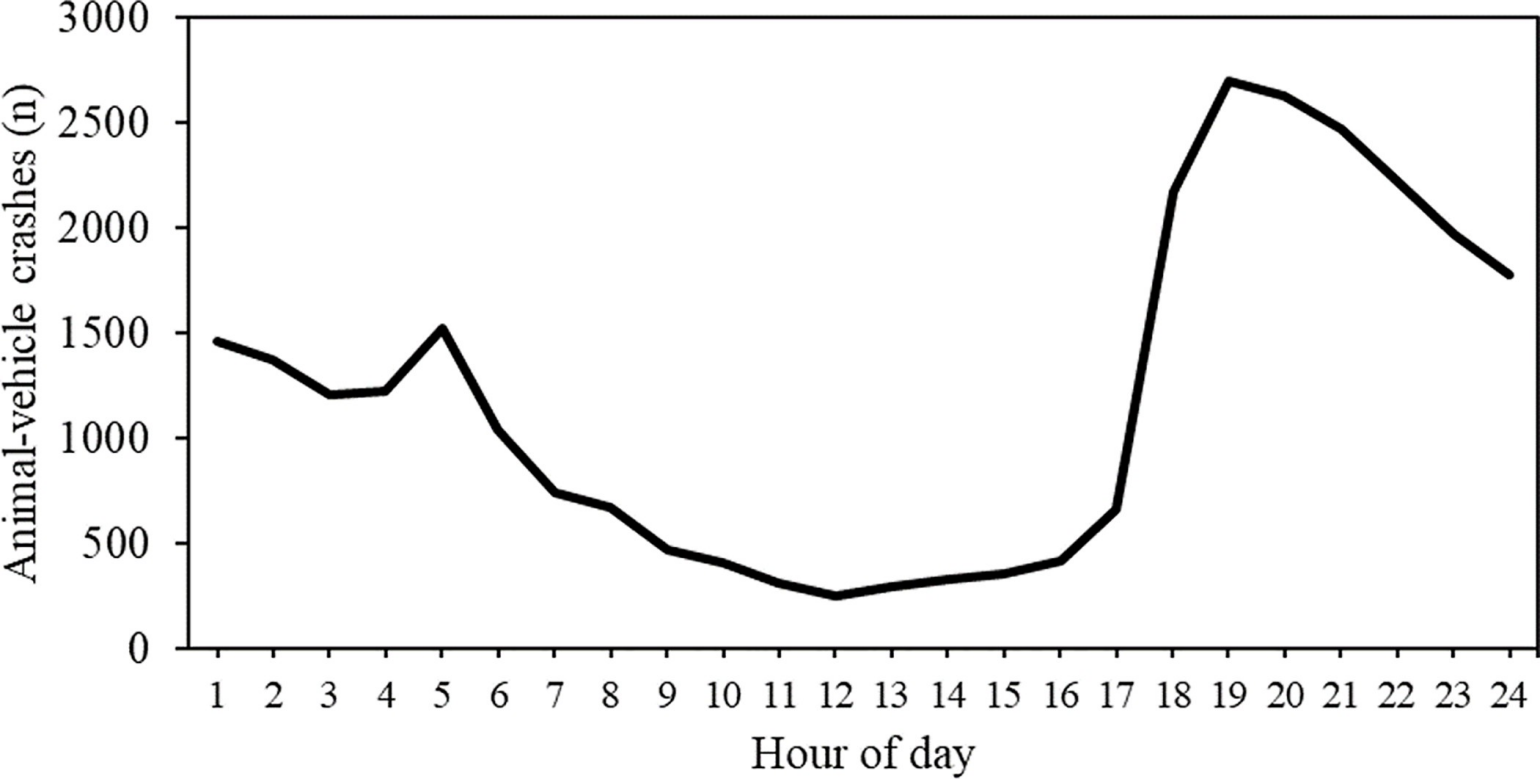
Total number of animal-vehicle crashes (2003–2013) by hour of day on both private and public state roads in São Paulo State.

Of the 28,724 crashes, 71,62% (n = 20,575) occurred at night between the hours of 19:00h and 6:00h, with peaks around dawn and dusk (Fig 5).

### Costs of animal-vehicle crashes to society

The total annual costs to society associated with the reported animal-vehicle crashes in São Paulo State was R$ 56,550,642 (US $ 25,144,794 in 2013 [23]. The average cost for an animal-vehicle crash, regardless of whether human injuries and fatalities occurred, was R$ 21,656 (US $ 9,629). The average cost for an animal-vehicle crash without human injuries or fatalities (82% of the 23,415 animal-vehicle crashes) was almost 50% lower; R$ 11,364 (US $ 5,053), whereas the average cost for a crash with human injuries or fatalities (18% of the animal-vehicle crashes) was R$ 67,048 (US $ 29,813). However, most of the costs (57%) were associated with crashes that involved human injuries or fatalities.

### Legal perspectives and financial compensation

**Fig 6:**
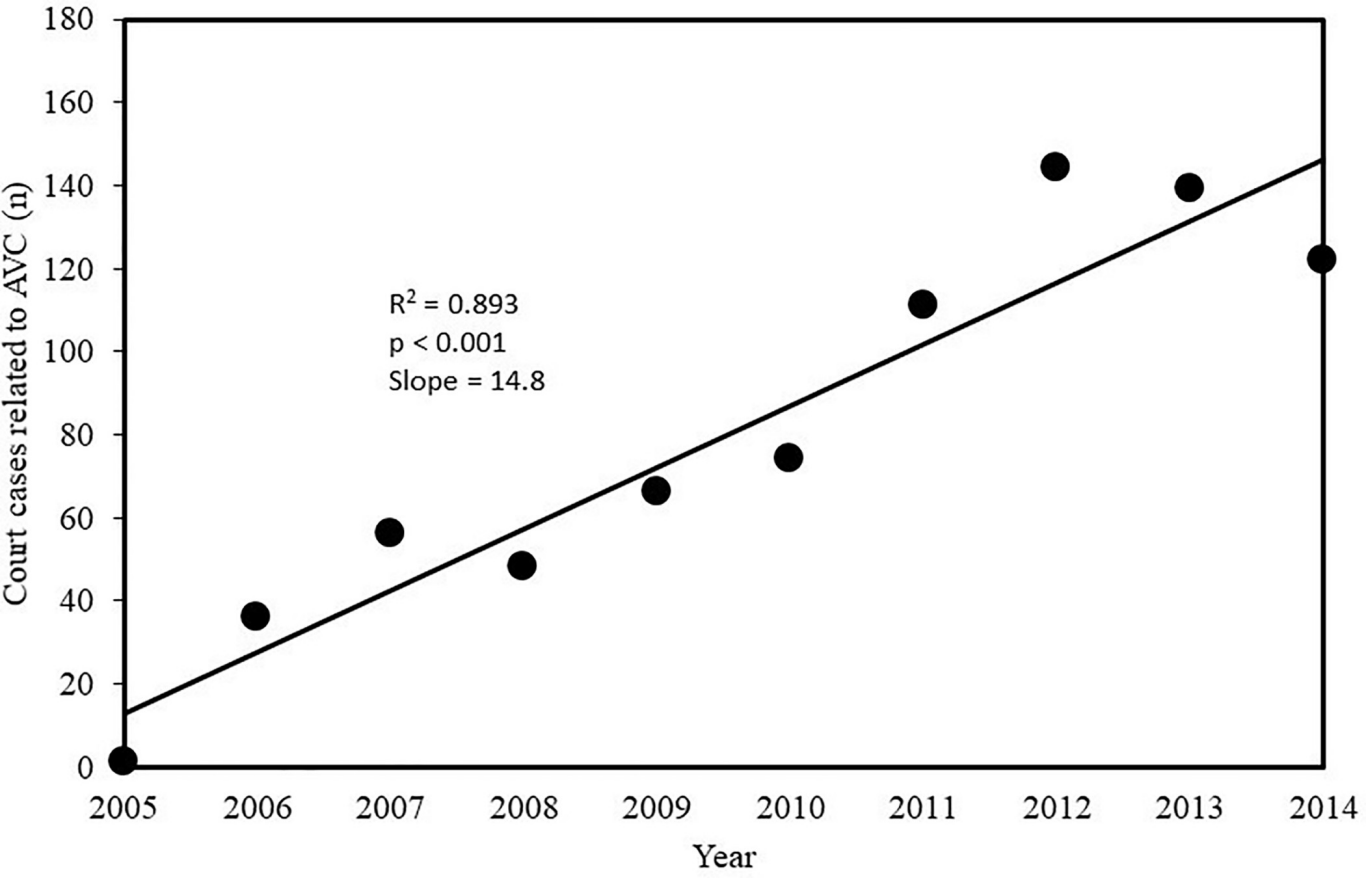
Number of court cases per year related to animal-vehicle collisions at the Court of Appeal of the State of São Paulo (TJSP–Tribunal de Justiça do Estado de São Paulo—2° instância).

The number of court cases related to animal-vehicle collisions increased substantially between 2005 and 2014 (Fig 6; S4 Table). The average increase in court cases was 14.8 per year. Of the 797 court cases, 14.4% included human fatalities (n = 115 in total, mean = 11.5 per year). Most of the court cases (69.5%, n = 554) were related to animal-vehicle collisions on roads managed by toll road companies, whereas 30.1% (n = 240) were on state roads managed by DER, and only 0.4% (n = 3) on city roads.

**Fig 7:**
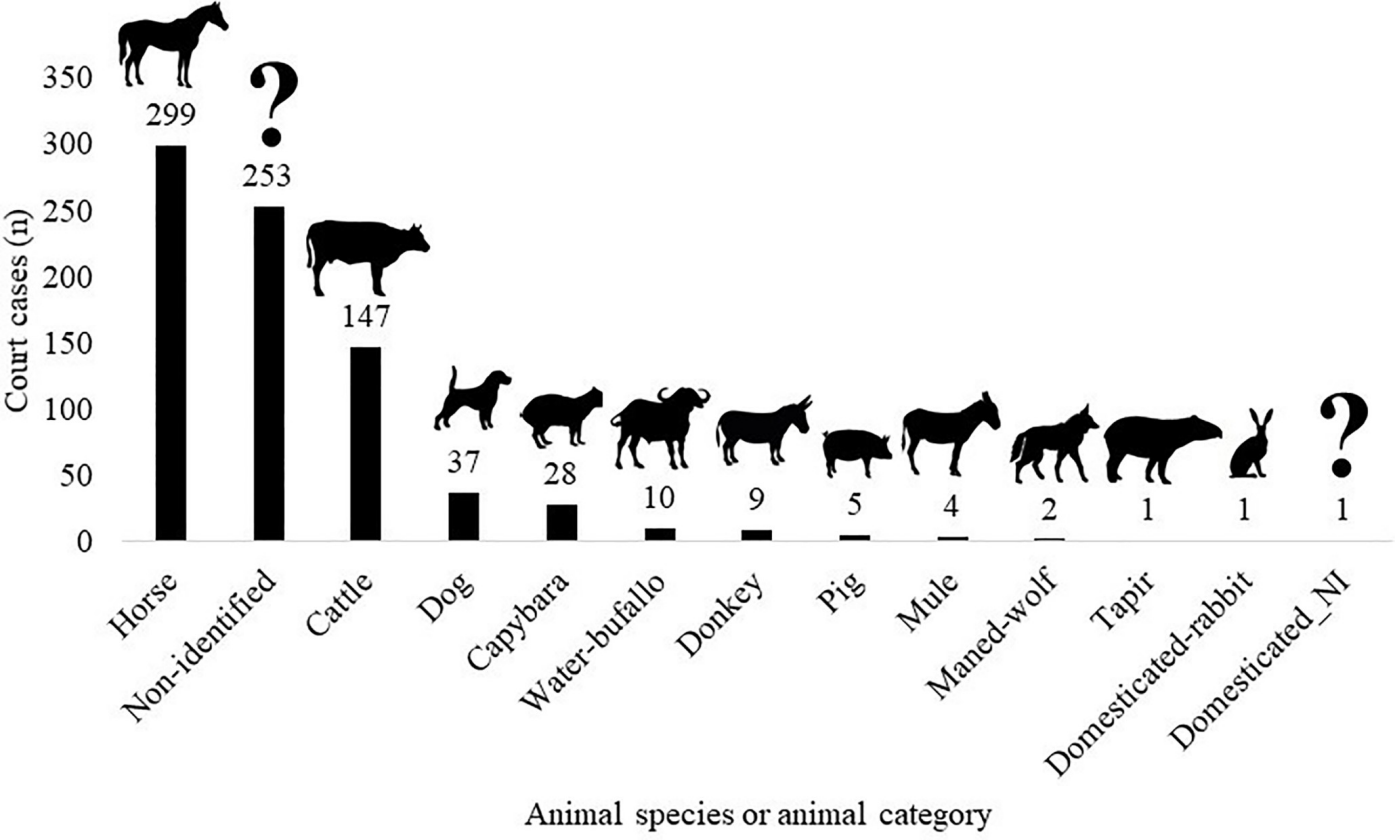
The animal species or animal group mentioned in the court cases related to animal-vehicle collisions in São Paulo State (2005–2014).

Most of the court cases related to collisions with domesticated species (64.4%, n = 513), followed by non-identified animal species (31.8%, n = 253), and wild species native to Brazil (3.9%, n = 31) (Fig 7, S4 Table). Horses (37.5%, n = 299), cattle (18.4%, n = 147), and dogs (*Canis lupus familiaris*) (7.2%, n = 37) were the most frequently reported species in the court cases (Fig 7). The most frequently reported wild animal species was capybara (3.5%, n = 28), followed by maned-wolf (*Chrysocyon brachyurus*) (0.3%, n = 2) and lowland-tapir (0.1%, n = 1).

Of the 797 court cases, the vast majority (91.7%, n = 731) were awarded in favor of the plaintiffs with associated financial compensation. The court records indicated that the awards in favor of the plaintiffs were based on Article. 37, §6 of the Brazilian Federal Constitution [30] and the Code of Consumer Protection [31].

Brazilian Federal Constitution, 1988

“Article 37.°The governmental entities and entities owned by the Government in any of the powers of the Union, the states, the Federal District and the Municipalities shall obey the principles of lawfulness, impersonality, morality, publicity, and efficiency, and also the following: (CA No. 19, 1998; CA No. 20, 1998; CA No. 41, 2003; CA No. 42, 2003; CA No*.* 47, 2005)”

“§6.°Public legal entities and private legal entities rendering public services shall be liable for damages that any of their agents, acting as such, cause to third parties*,* ensuring the right of recourse against the liable agent in cases of malice or fault.”

Brazilian Consumer Defense Code (Federal Law n° 8.078/1990)

“Art. 2*.* A consumer is any physical person or corporate entity who acquires or uses a product or service as a final user.”

“Art. 14*.* The service supplier will be responsible, regardless of the existence of guilt, for providing the necessary reparations for the damage caused to consumers due to any defects pertaining to service provision, as well as for insufficient or inadequate information about the nature of the service and the risks involved.”

“Art. 22. Public agencies, by themselves or through their companies*,* service providers, or any other form of entrepreneurship, will be required to provide products that are adequate, efficient, safe and, regarding the essential, continuous.”

“Sole paragraph. In the case that the obligations mentioned in this article are not followed, totally or partially, corporate entities will be compelled to obey them and provide reparations for any damages caused*,* in accordance to what is set forth by this Code.”

Only 8.3% (n = 66) of the court cases were awarded in favor of the road administrator, and financial compensation to the plaintiffs was denied. Just over half of the cases (51,5%, n = 34) in which financial compensation was denied to the plaintiff suffered from procedural problems, including lack of sufficient evidence, ineptitude of the initial petition for the case, or the case was time barred. For the remaining 32 cases (48.8%), the judge ruled that the road administrator could not be held legally responsible for animals entering the road. The judge considered these animal movements to be fortuitous, and, as a consequence, the animal-vehicle collisions could not necessarily be prevented, at least not along the entirety of the road network.

**Fig 8:**
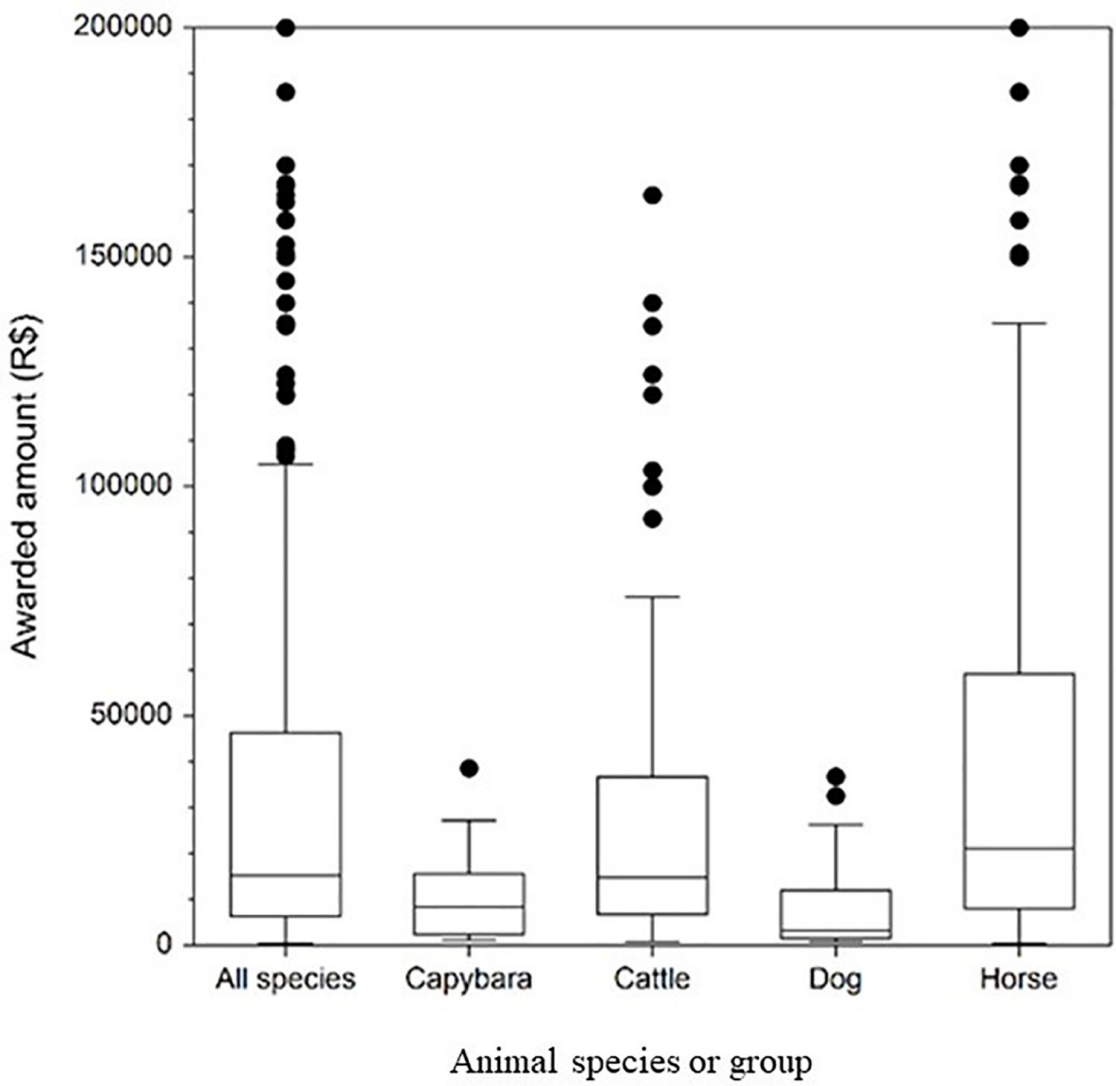
Box plot of the amount awarded to the plaintiffs by animal species. Box: middle 50% of the data (25^th^-75^th^ percentile); horizontal line: median; whisker boundaries: 1.5 times inter-quartile range; dots: outliers.

Between 2005 and 2014, a total of R$ 24,633,798 (US $ 10,050, in 2014) [23] was awarded to the plaintiffs. On average, the defendants spent R$ 2,463,380 (US $ 1,005,051) per year compensating the plaintiffs. The average value awarded in a court case related to an animal-vehicle collision, regardless of the animal species, was R$ 43,452 (US $ 17,728) (n = 578 court cases with a known awarded value). For capybara-vehicle collisions the average value awarded was R$ 10,888 (US $ $4,442) (n = 22 court cases with a known awarded value), cattle was, R$ 32,511 (US $ 13,462, n = 110), dog was R$ 8,151 (US $ 3,326, n = 24), and for horse was R$ 52,563.62 (US $ 21,446, n = 234) (Fig 8).

## Discussion

The crash data from the Military Highway Police of São Paulo State (PMRSP) showed that the number of reported animal-vehicle crashes are increasing and represent 3.3% of the total number of reported crashes in São Paulo State. This percentage is higher than the national crash data in Brazil (1.9%; [19]), but lower than the national crash data in the United States (about 4–5%; [13], [12]). An increase in traffic volume, new roads in remote areas, and increasing populations of species that adapt to living in agricultural and residential areas are all likely contributing factors to the increase of the reports.

In São Paulo State, 18.5% of the reported animal-vehicle crashes resulted in human injuries or fatalities. This is similar to the rate for human injuries for crashes with very large mammal species (e.g. moose) in the United States [17], but lower than the 40.9% for the national crash data in Brazil [19]. These data suggest that the national animal-vehicle crash data in Brazil are restricted to the more severe cases, at least when compared to the animal-vehicle crash data in São Paulo State. The data also suggest that the reported animal-vehicle crashes in São Paulo State have a human injury rate (40.9% of the reported animal-vehicle crashes) that is substantially higher than the United States (2.8–9.7% of reported deer-vehicle crashes and about 18–23% for larger species such as moose) [17], [19]. The court cases related to animal-vehicle crashes in São Paulo State predominantly dealt with domesticated horses and cattle, rather than deer that dominate the reported wildlife-vehicle crashes in the United States [17]. The high rate of human injuries for animal-vehicle crashes in São Paulo State may also be related to the high percentage of motorcycles (19.2%) compared to the United States (3.2% motorcycles). [32], [33]. Animal-vehicle crashes with lighter vehicles, especially motorcycles, are more likely to result in human injuries and fatalities because the driver is easily launched from a motorcycle and not protected from direct physical impact by the “protective cage” associated with passenger cars and heavier vehicles. For example, the Center for Disease Control and Prevention [34] in USA reported relatively few non-fatal hospitalizations from motorcycle-animal collisions, which suggests that a motorcycle crash with an animal is likely to be fatal to the operator of the motorcycle. Other factors that may contribute to the relatively high rate for human injuries in Brazil are the potentially low presence and use of seatbelts and airbags [35], narrow lanes and road shoulders, other road design characteristics that present a relatively high risk to human safety, dangerous driving styles, and the comparatively slow response time of emergency services. Note that air bags have been standard equipment in all new passenger cars in the United States since 1998. However, airbags did not become mandatory for new vehicles in Brazil until 2014 [32].

Though the total number of animal-vehicle crashes was similar for roads managed by toll road companies and DER (public state roads), the number of animal-vehicle crashes with human injuries or fatalities was much higher on roads managed by DER. This is most likely because DER has fewer modern roads with a safer road design, slower medical and mechanical assistance and lower presence of Military Highway police than toll roads. Interestingly, while toll roads represent about one third of all paved roads in São Paulo State, they received most of the reported animal-vehicle crashes (52%) [36]. This is probably related to the relatively high traffic volume, high vehicle speed, and relatively high night-time use compared to DER roads. While the number of animal-vehicle crashes with human injuries or fatalities was much higher on roads managed by DER than roads managed by toll road companies, 62.5% of the court cases related to animal-vehicle collisions on toll roads. Furthermore, there is no difference concerning the liability between private and public road administrators for animal-vehicle collision, and the traveling public is more likely to sue toll road companies than public road administrators. This may be related to the toll fee that needs to be paid each time when using a toll road; the act of paying may make the roles of a service provider and a consumer more current and explicit.

There were slightly fewer crashes during the wet season (October-March) compared to the dry season (April-September) when animals generally expand their range in search of food [37]. However, there was relatively little variation in the number of animal-vehicle crashes over the course of a year in the tropical and sub-topical region of São Paulo State compared to temperate regions with greater seasonal differences in weather and associated differences in animal behavior such as a more defined mating season with higher activity [11], [38], [12]. The weak seasonal pattern in animal-vehicle crashes in São Paulo State may also be due to the crash data from the Military Highway Police of São Paulo State which were based on a combination of domestic and wild species. Our analysis identified capybara as the wild mammal species most frequently involved in collisions in São Paulo State. The capybaras do not have a distinct mating season [39], which contrasts with white-tailed-deer (*Odocoileus virginianus*) which is the wild mammal species most frequently hit in the United States. White-tailed deer have a distinct mating season that is associated with relatively high occurrence of collisions [12], [40]. However, the time of day during which most animal-vehicle collisions occurred was similar to that in temperate regions; predominantly in the dark, especially around dusk till midnight, and around dawn [11], [12].

Costs associated with animal-vehicle crashes increased during the time frame this study was carried in São Paulo State. While crashes with human injuries or fatalities only represented 18% of the animal-vehicle crashes, they accounted for the majority of the costs (57%). This suggests that, from both a human safety and a financial perspective, efforts to reduce animal-vehicle collisions should not only be directed at reducing the number of animal-vehicle crashes, but also their severity.

From a legal perspective, road administrators (private or public) are usually (91.7% of the cases) held responsible for animal-vehicle collisions. Therefore, in São Paulo State, toll road companies or DER are typically required to provide monetary compensation for the damages and losses to the plaintiffs in court cases related to animal-vehicle collisions. On average, the defendants spent R$ 2,463,380 (US $ 1,005,051) per year compensating the plaintiffs. However, since animal-vehicle crashes have been increasing, the costs have likely been substantially higher in recent years. The awarded amounts were highest for large domesticated species such as horses and cattle.

Road administrators are generally held responsible for animal-vehicle collisions as they provide a service to the traveling public and are obligated to protect their customers. This type of liability does not suggest actual negligence or intent to harm on the part of the road administrators [41], rather, it is based on protecting the “customer” who is considered the more vulnerable of the two parties. The view of the courts is that the recipient of the service (the traveling public) can expect safe transportation. If an animal (wild or domesticated) enters the roadway and is involved in a vehicle collision, the road administrator is considered to be non-compliant regarding the obligation to provide safe travel, free and unimpeded. Note that if the owner of a domesticated animal is known and found, the road administrator can sue the owner of the domestic animal. In these select cases, both the road administrator and the owner of the domestic animal can be held legally responsible for the collision. However, the owner is typically unknown or hard to find, and most law suits only involve the road administrator. Similarly, in Italy, the road administrator is held liable for wildlife-vehicle collisions [42], where the legal base is similar to Brazilian legal system. In Italy, when a collision involves a domesticated animal, and if the owner is known, the owner is held responsible. However, if there is no known owner of the domesticated animal, the municipality where the animal was hit is held responsible as the municipality failed to prevent the abandonment of the animals and exposed the road users to a risk. Legal responsibility for animal-vehicle collisions is different in other countries. For example, in Spain animal-vehicle collisions are considered the driver’s responsibility, but the road administrator may be liable if the road was poorly signed (e.g. few animal crossing signs) or if there was no or poor maintenance of the fences [43]. In the United Kingdom, liability for animal-vehicle collisions with domesticated animals lie with the animal’s owner. If the animal has no owner, there is no responsible party, and thus no possibility for compensation [44]. In comparison, in the United States, the responsibility for animal-vehicle collisions are typically with the driver, damages are usually covered by insurance companies and, in general, the road administrator assumes no responsibility and pays no compensation to the victims [45], [46].

The Brazilian legal system overwhelmingly holds the road manager liable for animal-vehicle collisions, both with wild and domestic species. The logical conclusion is that the Brazilian legal system expects road administrators to keep animals off the road. This then suggests that road administrators are expected to fence the entire length of the road system. While the standard right-of-way fences are typically a barrier for large livestock species (e.g. cattle, horses), they are not an absolute barrier for most wild animal species because right-of-way fences tend to be more permeable for wild species. Since road administrators are also expected to prevent wild animals from accessing the road, the Brazilian jurisprudence suggests that the right-of-way fences should also be an impermeable barrier to wild animal species. Whilst such fences would benefit human safety and reduce unnatural mortality for the animals, it would also increase the barrier effect of roads for wildlife [47]. In fact, it has the potential to make roads into an absolute barrier for wildlife, resulting in extreme habitat fragmentation, reduced population viability, and eventually the loss of many species from the landscape if no safe crossing opportunities are available.

In addition to the laws that relate to animal-vehicle collisions, Brazil also has an environmental licensing system for road construction and road upgrades [48]. The environmental licensing process can result in the requirement of mitigation measures aimed at reducing direct road mortality and providing safe crossing opportunities for wildlife. In practice, in Brazil and specifically in São Paulo State, such requirements are largely placed on toll road companies and rarely on DER. As a result, the toll road companies, especially in São Paulo State, are the leaders in the development and implementation of wildlife mitigation measures along highways in Brazil. Nonetheless, the requirements that result from the environmental licensing system, based on Environmental Impact Assessments or other instruments, are largely focused on biological conservation rather than human safety, and not on reducing collisions with large domestic species such as horses and cattle.

It appears that the Brazilian law regarding animal-vehicle collisions is focused on human safety while the environmental licensing system is largely focused on biological conservation. This results in a mismatch; the required mitigation measures for wildlife do not adequately match the legal responsibility for animal-vehicle collisions, and the legal responsibilities regarding animal-vehicle collisions do not adequately meet biological conservation requirements. In addition, the reality is that the legal system for animal-vehicle collisions in São Paulo State mostly deals with collisions involving domesticated species (64.4%) rather than wild animal species (3.9%). This results in further disconnection between the Brazilian legal system for animal-vehicle collisions, the environmental licensing process, and the need to not only improve human safety but also to address the impacts of roads and traffic on the environment, including habitat connectivity for wildlife. Is important to highlight that 31.8% of the court cases were with non-identified animal species. This may be related to the fact that police officers (non-experts) are not able to identify many wild animal species, and that the actual percentage of court cases involving collisions with wild animal species is much higher [49].

Abandoned domesticated animals are very common in Brazil, but there is no specific policy that addresses the problem. Low income people in rural areas are known to abandon old or injured horses and dogs so that they do not have to deal with the financial expenses associated with veterinary care (Osnir Ormon Giacon, Concessionária CART, pers. comm.). In addition, cattle and horses often enter the road corridor because the right-of-way fences are not present or poorly maintained. Many of these animals end up living on and alongside roads where they pose a serious threat to the traveling public. These abandoned animals can also pose a threat to wildlife, through the spread of diseases and predation (e.g. predation by feral dogs and cats) [50], [51].

In some areas of the western United States, especially in areas with low human population density, there are still open range livestock laws in effect. In selected areas, livestock (e.g. cattle), can roam freely, regardless of land ownership. Land owners, including road administrators, who want to keep livestock off their property, are required to fence livestock out. This is the opposite to the more common situation where the owner of livestock is required to fence in their animals and keep them off property owned by others [52].

While the Brazilian legal system overwhelmingly holds the road administrators responsible for animal-vehicle collisions (91.7% of the cases), there is a small percentage of cases (8.3%) where compensation to plaintiffs was denied. In these select few cases, the judge ruled that the road administrator could not be held legally responsible for animals entering the road because these animal movements were fortuitous, and, as a consequence, animal-vehicle collisions could not necessarily be prevented, at least not along the entirety of the road network. While it is true that wildlife-vehicle collisions can be widespread, their location is not necessarily random. Hotspots (concentrations of wildlife-vehicle collisions) can even be predicted based on a range of landscape and road variables (e.g. [53], [54], [55]). Hotspots maps based on monitoring data and maps resulting from predictive modelling can help with the planning and decision process for the potential implementation of mitigation measures aimed at reducing wildlife-vehicle collisions and at providing safe crossing opportunities for wildlife. Several tools have been developed for this specific purpose (e.g. [56], [57], [58], [59], [60]). This means that society has the knowledge and tools to identify and prioritize road sections that have a concentration of wildlife-vehicle collisions and that action can be taken to substantially reduce this problem. For major highways with a high design speed and a high traffic volume, the most effective and robust measure aimed at reducing wildlife-vehicle collisions and providing safe crossing opportunities for wildlife is erecting wildlife fences in combination with underpasses and overpasses [61], [62]. It is critical that the design of the fences and crossing structures matches the biological characteristics and requirements of the target species. While the design of the crossing opportunities such as underpasses and overpasses should allow for a live span of about 70 years, fences require more regular maintenance and may last only for about two decades [17]. Fence inspections and repair should be integrated into daily road maintenance practices. Fences that are not an effective barrier to the target species put humans and animals at risk and jeopardize the investments in the mitigation measures including the crossing structures.

Implementation of mitigation measures for wildlife species can even make economic sense [17], [20]. Livestock-vehicle collisions can be reduced by obligating livestock owners and road administrators to fence roads effectively and keep livestock off the highway. Abandonment of domesticated animals including horses, cattle and dogs is more difficult to address. This may require different norms in society and a greater awareness of the potential consequences of abandoning animals, not only regarding animal welfare, but also with regard to human safety.

To address some of the challenges mentioned above, Campos et al. [63] suggested the following management actions: (1) Informing people about diseases transmitted by free ranging dogs and cats, (2) Educating people about local biological diversity, (3) Making it illegal for people to abandon and feed (feral) domesticated species, (4) Establishing a deadline after which a removal program of free-ranging animals, specifically for dogs and cats, would be initiated (e.g. adoption or euthanasia programs).

## Conclusion

Our study demonstrates that the number of reported animal-vehicle crashes and associated costs are increasing in São Paulo State. Animal-vehicle crashes with large animal species, wild or domestic, are dangerous to people, especially for occupants of light vehicles such as motorcycles. Interestingly, Brazilian law holds road managers responsible for animal-vehicle collisions. This suggests that road managers have the obligation to keep animals, wild and domestic, off the highway. We suggest a better coordination between the laws that relate to animal-vehicle collisions and human safety, and the process for environmental licenses that focuses on both reducing collisions with wildlife and maintaining or improving habitat connectivity. These actions should ultimately result in a road system with increased human safety, reduced unnatural mortality for both domestic and wild animal species, safe crossing opportunities for wildlife, and reduced monetary costs to society. Our analyses of animal-vehicle collisions in São Paulo State goes beyond the traditional perspective of road ecology papers that mostly focus on biological conservation. Our study is a first step to specifically address human safety, economic and legal issues associated with animal-vehicle collisions in Brazil. The results can be valuable in the decision process for the planning of the construction or reconstruction of roads. It would take human safety, wildlife, as well as monetary costs into account. We believe that this is better than accepting a continuing increase in animal-vehicle collisions, and associated impacts on human safety, economic parameters, and the environment.

## Supporting information

S1 Text Clarification for Salário mínimo and Pensão vitalícia. (DOCX)

S1 Table Cost components for animal-vehicle crashes (based on [19]). (DOCX)

S2 Table Average costs for different categories of human injuries and human fatalities associated with a crash for each vehicle type (based on [19]). (DOCX)

S3 Table Maximum number of occupants for each vehicle type. (DOCX)

S4 Table Court cases in São Paulo State per year, type of road, type of animal involved in the crash and the awarded amount to the plaintiff. (DOCX)
