# Supplementary material for: Pay or prevent? Human safety, costs to society and legal perspectives on animal-vehicle collisions in São Paulo state, Brazil
Source: PLoS One. 2019 Apr 11;14(4):e0215152. doi: 10.1371/journal.pone.0215152 (PMC6459512; doi:10.1371/journal.pone.0215152)
Supplement: S1 Text — (DOCX) [file pone.0215152.s001.docx]

**S1 Text. Clarification for Salário mínimo and Pensão vitalícia**

**Salário Mínimo**

Brazil does not have a minimum hourly wage, but instead a minimum monthly salary (salário mínimo). Any regular worker (as opposed to a daily or hourly worker) must receive at least this minimum monthly salary from his or her employer. The value of the minimum salary is defined by a national law and is reassessed annually based on changes to the cost of living. Municipalities can choose to implement a higher minimum salary, but they cannot reduce the amount below the federal monthly rate. Under current Brazilian law, the minimum salary may be used to calculate minimum pensions for some retirees and older adults (e.g. their pension cannot be below the minimum salary), and/or to certain other people who are not employed.

**Pensão vitalícia**

Brazilian courts can award a life pension (pensão vitalícia) in cases where a person is disabled or killed because of the actions of another party. Life pension can be awarded to the person who is disabled or to the dependents of a person who is killed. Calculations of life pension are subjective and depend on a variety of both compensatory and punitive factors which may include, for example, the age of the dependent(s), average salary of the injured party (and/or minimum salary), potential earnings (thus potential lost earnings) of the injured party, and punitive damages. Once awarded, a life pension is then paid monthly for the remainder of the life the person to whom it is awarded (or the remainder of the childhood of child dependents (until age 18 or 21 depending on current law).
