# Supplementary material for: Pay or prevent? Human safety, costs to society and legal perspectives on animal-vehicle collisions in São Paulo state, Brazil
Source: PLoS One. 2019 Apr 11;14(4):e0215152. doi: 10.1371/journal.pone.0215152 (PMC6459512; doi:10.1371/journal.pone.0215152)
Supplement: S1 Table — (DOCX) [file pone.0215152.s002.docx]

**S1 Table.** **Cost components for animal-vehicle crashes (based on [19]).**

|  |  | **With human victim** | | |
| --- | --- | --- | --- | --- |
|  | **No human**  **victim (R$)** | **Minor injury (R$)** | **Severe Injury**  **(R$)** | **Fatality (R$)** |
| **Cost components** |  |  |  |  |
| **Total people cost** | **1086,14** | **8469,44** | **125133,91** | **433286,69** |
| Pre-hospital care | 4,42 | 759,18 | 1.111,73 | 86,28 |
| Hospital care | 625,60 | 5.661,76 | 72.855,40 | 143,19 |
| Post-hospital care | 40,59 | 208,5 | 3.150,21 | 0 |
| Loss of productivity | 415,53 | 1.840,00 | 47.797,94 | 432.557,99 |
| Transport of victim | 0 | 0 | 218,64 | 499,24 |
| **Total passenger car cost** | **7.159,12** | **12.126,82** | **12.126,82** | **19.323,91** |
| Removal | 193,22 | 168,1 | 168,1 | 743,6 |
| Material damage | 6.965,90 | 11.958,72 | 11.958,72 | 18.580,31 |
| **Total motorcycle cost** | **2.473,21** | **2.741,02** | **2.741,02** | **4.269,83** |
| Removal | 51,59 | 145,28 | 145,28 | 181,09 |
| Material damage | 2.421,61 | 2.595,74 | 2.595,74 | 4.088,74 |
| **Total bicycle cost** | **0** | **168,74** | **168,74** | **124,1** |
| Material damage | 0 | 168,74 | 168,74 | 124,1 |
| **Total pick-up truck cost** | **10.569,76** | **20.240,38** | **20.240,38** | **35.091,47** |
| Removal | 110,76 | 162,96 | 162,96 | 127,14 |
| Material damage | 10.396,71 | 19.846,39 | 19.846,39 | 34.861,81 |
| Commercial cargo loss | 62,29 | 231,03 | 231,03 | 102,51 |
| **Total truck cost** | **22313,92** | **65.656,01** | **65.656,01** | **47825,45** |
| Removal | 178,33 | 351,53 | 351,53 | 461,89 |
| Material damage | 18.805,75 | 57.009,43 | 57.009,43 | 41.718,38 |
| Commercial cargo loss | 3329,84 | 8295,05 | 8295,05 | 5645,19 |
| **Total bus cost** | **16069,3** | **10536,86** | **10536,86** | **20686,09** |
| Removal | 64,39 | 218,46 | 218,46 | 522,97 |
| Material damage | 16004,91 | 10318,39 | 10318,39 | 20163,12 |
| **Total other cost** | **10307,36** | **80108,63** | **80108,63** | **81209,29** |
| Removal | 88,52 | 177,05 | 177,05 | 1403,74 |
| Material damage | 10218,84 | 79931,58 | 79931,58 | 52522,13 |
| Commercial cargo loss | 0 | 0 | 0 | 27283,43 |
| **Inst. property cost** | **453,35** | **338,33** | **338,33** | **653,06** |
