# Supplementary material for: Pay or prevent? Human safety, costs to society and legal perspectives on animal-vehicle collisions in São Paulo state, Brazil
Source: PLoS One. 2019 Apr 11;14(4):e0215152. doi: 10.1371/journal.pone.0215152 (PMC6459512; doi:10.1371/journal.pone.0215152)
Supplement: S2 Table — (DOCX) [file pone.0215152.s003.docx]

**S2 Table. Average costs for different categories of human injuries and human fatalities associated with a crash for each vehicle type (based on [19]).**

| **Type of AVC** | **Average costs (R$)** |
| --- | --- |
| Passenger car_no human victim | 8698,61 |
| Pickup truck_no human victim | 12109,25 |
| Motorcycle_no human victim | 4012,70 |
| Bicycle_no human victim | 1539,49 |
| Truck_no human victim | 23853,41 |
| Bus_no human victim | 17608,79 |
| Other_no human victim | 11846,85 |
| Passenger car_minor human injury | 20934,59 |
| Pickup truck_minor human injury | 29048,15 |
| Motorcycle_minor human injury | 11548,79 |
| Bicycle_minor human injury | 8976,51 |
| Truck_minor human injury | 74463,78 |
| Bus_minor human injury | 19344,63 |
| Other_minor human injury | 88916,40 |
| Passenger car_severe human injury | 137599,06 |
| Pickup truck_severe human injury | 145712,62 |
| Motorcycle_severe human injury | 128213,26 |
| Bicycle_severe human injury | 125640,98 |
| Truck_severe human injury | 191128,25 |
| Bus_severe human injury | 136009,10 |
| Other_severe human injury | 205580,87 |
| Passenger car_human fatality | 453263,66 |
| Pickup truck_human fatality | 469031,22 |
| Motorcycle_human fatality | 438209,58 |
| Bicycle_human fatality | 434063,85 |
| Truck_human fatality | 481765,20 |
| Bus_human fatality | 454625,84 |
| Other_human fatality | 515149,04 |
