# Supplementary material for: Pay or prevent? Human safety, costs to society and legal perspectives on animal-vehicle collisions in São Paulo state, Brazil
Source: PLoS One. 2019 Apr 11;14(4):e0215152. doi: 10.1371/journal.pone.0215152 (PMC6459512; doi:10.1371/journal.pone.0215152)
Supplement: S3 Table — (DOCX) [file pone.0215152.s004.docx]

**S3 Table**. **Maximum number of occupants for each vehicle type.**

| Vehicle type | Max. Occupants (n) |
| --- | --- |
| Bicycle | 1 |
| Motorcycle | 2 |
| Passenger car | 5 |
| Pick-up truck | 5 |
| Truck | 2 |
| Bus | 46 |
| Other | 2 |
