# Supplementary material for: Pay or prevent? Human safety, costs to society and legal perspectives on animal-vehicle collisions in São Paulo state, Brazil
Source: PLoS One. 2019 Apr 11;14(4):e0215152. doi: 10.1371/journal.pone.0215152 (PMC6459512; doi:10.1371/journal.pone.0215152)
Supplement: S4 Table — (DOCX) [file pone.0215152.s005.docx]

**S4 Table. Court cases in São Paulo State per year, type of road, type of animal involved in the crash and the awarded amount to the plaintiff. NI = non-identified; n = number of individuals.**

| **Year** | **Nº cases** | **Human deaths (n)** | **NI Animals (n)** | **Domesticated Animals (n)** | **Wild Animals (n)** | **Public roads (n)** | **Toll roads (n)** | **City roads (n)** | **Awarded amount (R$)** |
| --- | --- | --- | --- | --- | --- | --- | --- | --- | --- |
| **2005** | 1 | 1 | 1 | 0 | 0 | 0 | 1 | 0 | 300.000,00 |
| **2006** | 36 | 6 | 15 | 21 | 0 | 14 | 22 | 0 | 916.311,75 |
| **2007** | 56 | 8 | 27 | 28 | 1 | 16 | 40 | 0 | 2.485.501,21 |
| **2008** | 48 | 7 | 16 | 31 | 1 | 14 | 33 | 1 | 761.131,48 |
| **2009** | 66 | 12 | 17 | 45 | 4 | 30 | 36 | 0 | 1.815.236,09 |
| **2010** | 74 | 11 | 24 | 49 | 1 | 27 | 47 | 0 | 2.468.316,78 |
| **2011** | 111 | 14 | 30 | 79 | 2 | 47 | 64 | 0 | 2.768.600,64 |
| **2012** | 144 | 22 | 34 | 105 | 5 | 39 | 105 | 0 | 3945.765,04 |
| **2013** | 139 | 24 | 45 | 82 | 12 | 31 | 107 | 1 | 4.622.442,06 |
| **2014** | 122 | 10 | 44 | 73 | 5 | 22 | 99 | 1 | 4.976.479,64 |
| **Total** | **797** | **115** | **253** | **513** | **31** | **240** | **554** | **3** | **25.059.784,69** |
